# Effects of dietary 25-hydroxycholecalciferol supplementation on growth performance, blood vitamin D and antioxidant status in nursery pigs

**DOI:** 10.5713/ab.25.0029

**Published:** 2025-05-12

**Authors:** Chan Ho Kwon, Eva S. Safaie, Savannah L. Locke, Jannell A. Torres, Zhaohui Yang, Xi Chen, Young Dal Jang

**Affiliations:** 1 Department of Animal and Dairy Science, University of Georgia, Athens, GA, USA; 2 Nutribins LLC, Covina, CA, USA

**Keywords:** 25-Hydroxycholecalciferol, Antioxidant Status, Growth Performance, Nursery Period, Vitamin D_3_, Weaning Pigs

## Abstract

**Objective:**

This study was conducted to evaluate the effect of dietary 25-hydroxycholecalciferol (25-OHD_3_) supplementation on growth performance, blood 25-OHD_3_ level, and antioxidant parameters in nursery pigs.

**Methods:**

A total of 48 newly weaned piglets (5.27±1.42 kg initial body weight) were allotted to 3 treatments in 4 replicates with 4 pigs per pen for a 28-d feeding trial in two phases for d 0–14 (Phase 1) and d 14–28 (Phase 2) postweaning with basal diets without supplemental vitamin D_3_ (VD_3_). Treatments were: 1) basal diet+2,000 IU/kg VD_3_ supplementation, 2) basal diet+1,000 IU/kg 25-OHD_3_ supplementation, and 3) basal diet+2,000 IU/kg 25-OHD_3_ supplementation. Growth performance, plasma 25-OHD_3_ and malondialdehyde (MDA) levels, total antioxidant capacity, and superoxide dismutase activity were measured.

**Results:**

There was no significant difference in growth performance among dietary treatments until d 21 postweaning. Dietary 25-OHD_3_ supplementation increased feed intake (p<0.07) and growth rate (p<0.05) greater than the VD_3_ treatment in d 21–28 and d 14–28 postweaning, resulting in a greater growth rate in the overall period (p = 0.10). Increasing levels of 25-OHD_3_ supplementation resulted in greater plasma 25-OHD_3_ concentrations at d 14 and 28 postweaning (p<0.05), while decreasing plasma MDA levels at d 28 postweaning (p<0.05) with no differences in plasma superoxide dismutase activity and total antioxidant capacity. In the broken-line analysis, the estimated plasma 25-OHD_3_ concentration for plasma MDA concentration to reach the minimum level was 23.7 ng/mL (p<0.05).

**Conclusion:**

Supplementing 25-OHD_3_ in nursery diets increased blood vitamin D status and had potential to enhance feed intake and growth rate in the late nursery period, while reducing oxidative stress compared with VD_3_ supplementation.

## INTRODUCTION

Weaning is one of the most stressful periods separating piglets from their sow as it can negatively affect the immune system, intestinal function, and cause oxidative stress in pigs [1–3]. Vitamin D, a fat-soluble vitamin, plays an important role in bone development, mineralization, and other biological functions, including immune and antioxidant systems [4,5]. Therefore, supplementing diets with vitamin D is critical for health, growth and development of weaning pigs as a previous study reported that an increased vitamin D status for weaning pigs may promote intestinal robustness against disease challenge [6]. Generally, vitamin D_3_ (VD_3_) has been supplemented to swine diets as a traditional form because pigs are housed in confinement housing, thus limiting vitamin D synthesis in the body. The U.S. swine industry typically supplemented diets with VD_3_ at the levels ranging from 1,600 to 2,600 IU/kg for nursery pigs from weaning to 25 kg body weight [7]. Recently, 25-hydroxycholecalciferol (25-OHD_3_) has been increasingly used in swine diets as a circulating form of VD_3_ that is hydroxylated in the liver after absorption, which bypasses the metabolic process of VD_3_ in the liver [8,9]. The 25-OHD_3_ is known to have stronger biological activity than VD_3_ [10]. The intestine more easily absorbs 25-OHD_3_ than VD_3_ as its absorption is less dependent on bile acids and chylomicrons than VD_3_ [11]. In addition, vitamin D-binding proteins exhibit a 500 times stronger affinity for 25-OHD_3_ than VD_3_ [12] and greater daily excretion of metabolites was observed in VD_3_ than 25-OHD_3_ [13]. Previous studies reported that dietary 25-OHD_3_ supplementation improved growth performance, immunity, and antioxidant status in nursery pigs when supplemented to diets with certain levels (2,000–2,500 IU/kg) of supplemental VD_3_ [10,14]. While these studies investigated the efficacy of 25-OHD_3_ in increasing plasma 25-OHD_3_ levels and improving antioxidant capacity of weaned pigs, it is also important to clearly demonstrate the effect of dietary 25-OHD_3_ supplementation in diets without supplemental VD_3_ and its potential relationships with antioxidant capacity and oxidative stress in weaning pigs, as they are susceptible to oxidative stress after weaning, which can affect postweaning growth performance [15]. Therefore, this study was conducted to evaluate the effects of dietary VD_3_ and 25-OHD_3_ supplementation on growth performance, blood vitamin D status, and antioxidant parameters of weaned pigs.

## MATERIALS AND METHODS

### Animals, experimental design, and housing

At weaning, a total of 48 newly weaned pigs (Camborough× PIC337 and [Camborough×Berkshire]×PIC337; 5.27±1.42 kg initial body weight; weaned at 18.7±0.78 d of age) were allotted to 1 of 3 dietary treatments in 4 replicates with 4 pigs (2 barrows and 2 gilts) per pen based on body weight, breed, sex, and littermate in a randomized complete block design for a 28-d feeding trial. Treatments were: 1) basal diet with 2,000 IU/kg VD_3_ supplementation (Control), 2) basal diet with 1,000 IU/kg 25-OHD_3_ supplementation, and 3) basal diet with 2,000 IU/kg 25-OHD_3_ supplementation. Basal diets were prepared without supplemental VD_3_. All pigs were housed in nursery pens (1.0 m×2.0 m) with woven-wire flooring, fed *ad libitum*, and had free access to water in an environmentally controlled nursery facility at the University of Georgia Large Animal Research Unit. No creep feed was provided during the lactation period. The pigs were fed the same treatment diets with two diet phases, including d 0–14 postweaning (Phase 1), and d 14–28 postweaning (Phase 2). The 25-OHD_3_ product (SmartD) was obtained from Nutribins LLC (Covina, CA, USA).

### Experimental diets

All pigs were fed corn-soybean meal-based diets in mash form that were formulated to meet or exceed nutrient requirement estimates of NRC [16] for 7–11 kg (Phase 1) and 11–25 kg (Phase 2) of pigs (Table 1). The VD_3_ supplementation level in the control diet was similar to the average level commonly used in nursery diets within the U.S. swine industry and 25-OHD_3_ was supplemented in the diet at 1,000 or 2,000 IU/kg, equivalent to half or the full dose of VD_3_ used in the control diet. To minimize differences in non-treatment components of the diets, a basal diet was first mixed with a VD_3_-free vitamin premix and without the inclusion of corn starch. The basal diet was divided into 3 equal fractions. For each treatment, a premix containing either VD_3_ or 25-OHD_3_ was prepared by blending the respective vitamin D source with corn starch. The VD_3_ treatment diet was mixed by adding a premix providing 2,000 IU/kg of VD_3_. The two 25-OHD_3_ treatment diets were prepared by supplementing the remaining fractions with premixes providing either 1,000 or 2,000 IU/kg of 25-OHD_3_, respectively.

### Data and sample collection

The pigs were individually weighed at the start of the trial, d 7, 14, 21, and 28 post-weaning. The pen-based feed disappearance was measured when the pigs were weighed. Average daily gain (ADG), average daily feed intake (ADFI), and gain-to-feed (G:F) ratio were calculated. Blood samples (10 mL) were collected from eight pigs per treatment (2 pigs per pen; 1 barrow and 1 gilt) selected based on average body weight in each pen on d 14 and 28 post-weaning via jugular venipuncture in disposable vacutainer tubes containing the anticoagulant K_3_ EDTA (Becton Dickinson, Franklin, NJ, USA). Plasma samples were obtained by centrifugation at 2,500×g for 30 min at 4°C and stored at −80°C until analysis.

### Chemical analysis

Plasma samples were analyzed for 25-OHD_3_ at Heartland Assays (Ames, IA, USA), and antioxidant parameters including superoxide dismutase (SOD) activity, total antioxidant capacity (T-AOC), and malondialdehyde (MDA) levels using colorimetric kits (Cayman Chemical Company, Ann Arbor, MI, USA) and a spectrophotometer (Thermo Fisher Scientific, Waltham, MA, USA).

### Statistical analysis

All data obtained in the current study were analyzed in accordance with randomized complete block design using the PROC MIXED procedure of SAS (ver. 9.4; SAS Institute, Cary, NC, USA). A pen was used as an experimental unit for the analysis of growth performance data. An individual pig was used as an experimental unit for blood analyses. The models included the treatment as a fixed effect and the replicate as a random effect for growth performance and the replicate within pen and pen as random effects for blood parameters. A single degree of freedom contrast was performed to make comparison between VD_3_ treatment vs. combined 25-OHD_3_ treatments (1,000 and 2,000 IU/kg of 25-OHD_3_ treatments). The least square means were separated using the PDIFF option of SAS. Blood parameters were analyzed to detect any relationship between the levels of plasma 25-OHD_3_ and antioxidant parameters (T-AOC, SOD, and MDA) within each sampling day was performed using PROC REG in SAS. When significant quadratic correlations were observed, the linear broken-line regression analysis was performed using PROC NLIN to estimate plasma 25-OHD_3_ concentrations to reach the minimum or maximum plasma antioxidant parameters as described by Robbins et al [17]. Statistical differences were established at p≤0.05 and tendencies were established at p≤0.10.

## RESULTS

### Growth performance

The ADFI was significantly greater in both 25-OHD_3_ treatments than the VD_3_ treatments in d 21–28 postweaning (p< 0.05; Table 2). When the two 25-OHD_3_ treatments were combined, the pigs fed diets supplemented with 25-OHD_3_ had greater ADFI in d 21–28 (p<0.05) and d 14–28 postweaning (p = 0.07; tendency) than those fed diets supplemented with VD_3_. They also had greater ADG in d 21–28 (p<0.05) and d 14–28 (p<0.05) postweaning and overall period (p = 0.10; tendency) than those fed diets supplemented with VD_3_.

### Plasma 25-OHD_3_ concentrations

The pigs in both 25-OHD_3_ treatments had greater plasma 25-OHD_3_ concentrations than those in the VD_3_ treatment at d 14 and 28 postweaning (p<0.05; Table 3) in which 2,000 IU/kg 25-OHD_3_ treatment had 1.81 and 2.03 times greater levels (p<0.05) than 1,000 IU/kg 25-OHD_3_ treatment at d 14 and 28 postweaning, respectively.

### Antioxidant capacity and oxidative stress parameters

There were no significant differences among dietary treatments in the SOD activity and T-AOC (Table 4). Plasma MDA levels of the pigs decreased by dietary 25-OHD_3_ supplementation compared with the VD_3_ treatment (p<0.05) at d 28 postweaning in which 2,000 IU/kg 25-OHD_3_ treatment had significantly lower MDA levels than the VD_3_ treatment (p< 0.05), while 1,000 IU/kg 25-OHD_3_ treatment had an intermediate value.

**Figure 1:**
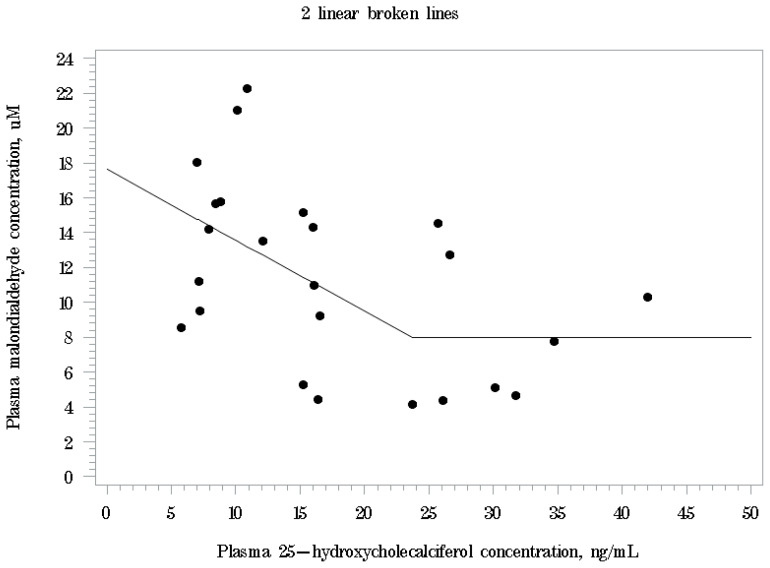
Broken-line analysis of plasma malondialdehyde (MDA) level by plasma 25-hydroxycholecalciferol concentrations at d 28 postweaning. The breakpoint was estimated at 23.7 ng/mL (p<0.05; 95% CI: [1.8–45.6 ng/mL]). The estimated regression equation was plasma MDA levels, μM = 7.98+0.407×(23.7–25-OHD_3_) if plasma 25-OHD_3_<23.7 ng/mL and plasma MDA level, μM = 7.98 if plasma 25-OHD_3_≥23.7 ng/mL. 25-OHD_3_, 25-hydroxycholecalciferol.

Linear and quadratic correlations were observed only between plasma 25-OHD_3_ and MDA levels at d 28 postweaning (linear: p<0.05, R^2^ = 0.231; quadratic: p<0.05, R^2^ = 0.265). Based on linear broken-line analysis, the estimated plasma 25-OHD_3_ concentration for plasma MDA level to reach the minimum level was 23.7 ng/mL (p<0.05; 95% CI: [1.8–45.6 ng/mL]; Figure 1). The estimated regression equation for plasma MDA levels (μM) is: MDA = 7.98+0.407×(23.7–plasma 25-OHD_3_) when plasma 25-OHD_3_ level is less than 23.7 ng/mL. When plasma 25-OHD_3_ level is greater than or equal to 23.7 ng/mL, plasma MDA level reaches its minimum at 7.98 μM.

## DISCUSSION

VD_3_ has been supplemented in nursery diets from 1,600 to 2,600 IU/kg [6] as VD_3_ supplementation may improve bone health, immune system, and antioxidant capacity of weaning pigs [10,18]. The 25-OHD_3_ has been considered as an alternative form of VD_3_ due to its relatively greater bioavailability than the VD_3_ in pigs, that can improve vitamin D status more efficiently than VD_3_ [9,14]. However, there is limited information on the direct comparison between VD_3_ and 25-OHD_3_ as sources of vitamin D, as several previous studies have focused on the effect of 25-OHD_3_ supplementation in diets that already contained VD_3_ at levels ranging from 2,000 to 2,500 IU/kg [10,14,19]. Therefore, the current study evaluated the effect of dietary 25-OHD_3_ supplementation in nursery pig diets containing no supplemental VD_3_ on growth performance, plasma 25-OHD_3_ concentrations, and antioxidant parameters compared with conventional VD_3_ supplementation.

In the current study, individual 25-OHD_3_ treatments showed no significant difference in growth performance from the VD_3_ treatment except that 1,000 or 2,000 IU/kg of 25-OHD_3_ supplementation increased feed intake in d 21–28 postweaning compared with 2,000 IU/kg of VD_3_ supplementation. A greater growth rate was observed in the late nursery and overall periods when the combined 25-OHD_3_ treatments were compared to the VD_3_ treatment. This result indicates that dietary 25-OHD_3_ supplementation had the potential to increase postweaning growth rate of piglets compared with dietary VD_3_ supplementation and this could be attributed to increased feed intake in the late nursery period by dietary 25-OHD_3_ supplementation. Although various factors affect postweaning feed consumption in pigs, one possible explanation for the increased feed intake could be due to the reduction in plasma MDA levels observed at d 28 postweaning in the current study when pigs consumed diets supplemented with 25-OHD_3_. This suggests that dietary 25-OHD_3_ supplementation may help reduce postweaning oxidative stress in pigs leading to increases in feed intake and growth rate, as oxidative stress could reduce postweaning feed intake and slow down weight gain of piglets [20]. Zhou et al [10] reported that 2,000 IU/kg of 25-OHD_3_ supplementation in the basal diets containing 2,000 IU/kg of VD_3_ had a greater growth rate and feed intake in the late nursery periods compared with 2,000 IU/kg VD_3_ supplementation in the basal diets. Zhang et al [14] also reported that additional 2,000 IU/kg of 25-OHD_3_ supplementation in the low Ca and P basal diets containing 2,500 IU/kg of VD_3_ for weaning pigs could increase growth rate in late nursery and overall periods compared with the basal diets with only 2,500 IU/kg of VD_3_. These findings are consistent with the current study, showing the potential of dietary 25-OHD_3_ supplementation to enhance feed intake and growth rate during the late nursery period. However, the current study lacked a significant difference in growth performance among the individual treatments with increasing levels of 25-OHD_3_ supplementation, which contrasts to the findings of the previous studies [10,14,18]. Although these previous studies utilized different breeds of pigs, diets with low Ca and P content, and varying levels of basal VD_3_ content, the reason remains unclear. However, possible explanations for this discrepancy could be that those previous studies used weaned piglets with higher initial weaning weights, ranging from 6.3 to 8.4 kg [10,14,18], compared to the 5.3 kg average in the current study and observed the effect of dietary 25-OHD_3_ supplementation only becoming apparent during the late nursery period. Therefore, with the results of the current study, it can be suggested that the growth response to 25-OHD_3_ supplementation may not become evident until the late nursery phase. In addition, Sandoval et al [21] reported no significant difference in growth performance when 1,000 or 2,000 IU/kg of 25-OHD_3_ was supplemented to the diets containing 44 IU/kg of VD_3_ for the pigs weighing from 10 to 38 kg. Therefore, the difference in initial weight could have influenced the response of pigs to dietary supplementation, potentially due to variations in body growth and development at different weaning weights [22]. Additionally, a sufficient duration of feeding 25-OHD_3_ may be necessary to observe more pronounced improvements in pig growth performance, as the effect was only seen during the late nursery period.

In the current study, there was no significant difference in feed efficiency among dietary treatments. This result agrees with previous studies [10,14,18] reporting that dietary 25-OHD_3_ supplementation up to 2,000 IU/kg did not affect feed conversion ratio. Therefore, this result indicates that dietary 25-OHD_3_ supplementation in nursery diets had potential to increase postweaning feed intake, thus growth rate in the nursery period instead of improving feed efficiency. However, further research may be needed to investigate the effect of dietary 25-OHD_3_ supplementation in nursery pigs under various conditions, such as different weaning weights, diet regimes, and feeding durations, in larger scale settings to explore the potential mechanisms through which it could enhance postweaning growth.

The 25-OHD_3_ is a circulating form of VD_3_ in the body, which has been commonly used as the best biomarker of body vitamin D status [23]. Moreover, concentrations of 25-OHD_3_ in different tissues are highly correlated with the blood 25-OHD_3_ concentrations [21,24]. In the current study, when pigs were fed the diets containing increasing levels of 25-OHD_3_ (1,000 and 2,000 IU/kg diet), plasma 25-OHD_3_ levels increased significantly compared with those fed diets with VD_3_. This result agreed with previous studies [14,19] reporting that dietary 25-OHD_3_ supplementation in nursery diets increased blood 25-OHD_3_ concentrations. Zhang et al [14] reported that the 2,000 IU/kg 25-OHD_3_ supplementation in low Ca and P nursery diets containing 2,500 IU/kg VD_3_ increased blood 25-OHD_3_ concentrations compared with the basal diets containing 2,500 IU/kg VD_3_ only. von Rosenberg et al [19] reported that plasma 25-OHD_3_ concentrations of pigs fed diets with 2,000 IU/kg of VD_3_ was 16.5 ng/mL after feeding treatment diets for 42 d, whereas the same level (2,000 IU/kg diet) of 25-OHD_3_ supplementation resulted in plasma 25-OHD_3_ concentrations 4 times greater (64.1 ng/mL) than the 2,000 IU/kg of VD_3_ diet, which was comparable to the results observed at d 28 postweaning in the current study. These results indicate that dietary 25-OHD_3_ supplementation is more effective in improving blood vitamin D status than the VD_3_ supplementation as 25-OHD_3_ is absorbed faster and to a larger extent than VD_3_ in the small intestine due to polarity and solubility [12,25] and VD_3_ should undergo the metabolic process to be converted to the circulating form (i.e., 25-OHD_3_) in the liver by 25-hydroxylase [9,26]. Although the current study only measured blood 25-OHD_3_ levels, von Rosenberg et al [19] also reported that increasing supplementation levels of 25-OHD_3_ increased tissue vitamin D status (liver, skin, and muscle) measured by tissue 25-OHD_3_ content. Similarly, Burild et al [24] reported that increasing supplementation levels of 25-OHD_3_ from 200 to 2,000 IU/kg diet increased tissue (liver, fat, and muscle) 25-OHD_3_ content in finishing pigs. Therefore, further study may be needed to accurately compare the bioavailability of these two VD_3_ sources by measuring both blood and tissue 25-OHD_3_ concentrations and finding a correlation between blood and tissue 25-OHD_3_ concentrations.

Interestingly, the 2,000 IU/kg of 25-OHD_3_ supplementation resulted in approximately 2 times greater plasma 25-OHD_3_ concentrations than the 1,000 IU/kg of 25-OHD_3_ supplementation, which follows a pattern consistent with a two-fold difference in their dietary supplementation levels. This result agreed with previous studies reporting that dietary 25-OHD_3_ supplementation in pig diets improved vitamin D status as determined by plasma 25-OHD_3_ concentrations in a dose-dependent manner [19,21]. von Rosenberg et al [19] reported that the pigs fed increasing supplementation levels of 25-OHD_3_ from 2,000 to 20,000 IU/kg diet for 42 d showed greater plasma 25-OHD_3_ concentrations than those fed the diet with VD_3_ at 2,000 IU/kg and the levels increased in a dose-dependent manner ranging from 64.1 to 360 ng/mL. Sandoval et al [21] also reported greater plasma 25-OHD_3_ concentrations in pigs fed diets with increasing levels of 25-OHD_3_ from 1,000 to 10,000 IU/kg diet for 39 d than the diet with 44 IU/kg VD_3_. As mentioned above, it is known that small intestine can absorb 25-OHD_3_ more efficiently and to a larger extent than VD_3_ due to its lesser dependence on bile acids and chylomicrons compared to VD_3_ [25,26] and it is also absorbed directly into portal blood as well as via chylomicrons into the lymph due to its polarity [26]. Therefore, these results indicate that plasma 25-OHD_3_ concentrations increase when 25-OHD_3_ is supplemented to diets in a dose-dependent manner.

Regarding antioxidant capacity and oxidative stress, the 2,000 IU/kg of 25-OHD_3_ supplementation reduced plasma MDA levels significantly compared with the 2,000 IU/kg of VD_3_ supplementation with intermediate levels in the 1,000 IU/kg of 25-OHD_3_ supplementation. This result agreed with a previous study that increasing 25-OHD_3_ supplementation levels in nursery diets decreased plasma MDA concentrations [10]. The plasma MDA level has been widely used as a marker of oxidative stress and lipid peroxidation to measure pigs’ health status. Weaning stress could decrease plasma SOD activity but increase plasma MDA levels in piglets, which may impact their postweaning feed intake, thus growth [15]. Vitamin D is thought to have antioxidant mechanisms related to inducing metallothionein synthesis which is known to have reactive oxygen species scavenger activities that can reduce oxidative stress [27,28] as 1,25-dihydroxycholecalciferol, a hormonally active form of VD_3_ that was activated from 25-OHD_3_ in kidney is known to increase mRNA expression of metallothionein gene [29]. Therefore, the result of the current study indicates that dietary 25-OHD_3_ supplementation could alleviate oxidative stress in weaning pigs and a higher level, which is 2,000 IU/kg diet, may potentially be more effective. In the broken-line analysis, although there is a relatively large variation observed due to individual pig values used in the analysis, plasma MDA level was minimized when the plasma 25-OHD_3_ concentrations reached 23.7 ng/mL, which could be achieved by approximately 1,600 IU/kg 25-OHD_3_ supplementation level when it is estimated by plasma 25-OHD_3_ concentrations in 1,000 and 2,000 IU/kg 25-OHD_3_ treatments at d 28 postweaning in the current study. However, because the 2,000 IU/kg of VD_3_ supplementation could not achieve this plasma 25-OHD_3_ level in nursery period, it may be beneficial to use 25-OHD_3_ as a vitamin D source in nursery diets due to its high bioavailability and effectiveness. Interestingly, although dietary 25-OHD_3_ supplementation increased plasma 25-OHD_3_ levels in a dose-dependent manner and reduced oxidative stress, greater supplementation level (2,000 IU/kg diet) did not result in further increase of growth rate and feed intake. Zhang et al. [18] reported that pigs fed diets with increasing levels of 25-OHD_3_ supplementation from 0 to 4,000 IU/kg showed quadratic responses in growth rate and feed intake with the greatest performance at the 2,000 IU/kg of 25-OHD_3_ supplementation level. However, in the current study, over 1,000 IU/kg 25-OHD_3_ may be needed to maximize its benefits for not only pig growth but also health including oxidative stress as the further reduction in plasma MDA levels was observed when the higher level of 25-OHD_3_ was supplemented to the nursery diets. Additionally, it should be noted that a further increase in dietary 25-OHD_3_ supplementation levels over 2,000 IU/kg might not result in additional benefits for growth and antioxidant status, as supplementing 25-OHD_3_ in nursery diets over 2,000 IU/kg may decrease growth rate, feed intake, and serum T-AOC activity of pigs [18]. Further studies are needed to identify the optimal plasma 25-OHD_3_ levels that maximize health benefits in pigs, particularly in terms of antioxidant capacity and oxidative stress, through varying levels of dietary 25-OHD_3_ supplementation. In addition, research should explore how baseline blood 25-OHD_3_ levels affect the effectiveness of 25-OHD_3_ supplementation in pigs.

Interestingly, the reduction of plasma MDA levels by dietary 25-OHD_3_ supplementation was only observed at d 28 postweaning but not at d 14 postweaning, although the increase in plasma 25-OHD_3_ concentrations was observed from d 14 postweaning. In previous studies [14,18], there were also inconsistent responses in these antioxidant parameters in pigs during the nursery period when they were fed diets supplemented with 25-OHD_3_. Zhang et al [18] reported an increase in serum T-AOC of nursery pigs at d 28 postweaning, but not at d 14 postweaning although the glutathione peroxidase (GSH-Px) activities and 25-OHD_3_ levels in serum increased at both d 14 and 28 postweaning. In addition, Zhang et al [14] also reported that serum SOD and catalase activities only increased at d 28 postweaning but not at d 14 postweaning when pigs were fed low Ca-P diets supplemented with 25-OHD_3_. Although there is no clear explanation in these responses, it can be noted that sufficient duration of feeding diets supplemented with 25-OHD_3_ may be needed to observe its effects on antioxidant status.

Although there was no effect of dietary 25-OHD_3_ supplementation on plasma SOD activity and T-AOC in the current study, previous studies reported that dietary 25-OHD_3_ supplementation at 2,000 IU/kg diet increased plasma T-AOC, SOD, and GSH-Px activities of weaned pigs [10,18]. Sauvé et al [30] reported that dietary 25-OHD_3_ supplementation to mycotoxin-contaminated diets for weaning pigs after lipopolysaccharide challenge did not improve catalase and SOD activities and MDA levels in blood, while increasing GSH-Px activity although there were different responses in mRNA expression of these antioxidant parameters in liver and jejunum. Therefore, the response of pigs to dietary 25-OHD_3_ supplementation in antioxidant parameters may vary depending on multiple factors such as status of oxidative stress, health challenge, environment, diets, etc. Due to these inconsistent results, further studies are also needed to demonstrate the efficacy of dietary 25-OHD_3_ supplementation on antioxidant capacity and oxidative stress markers in various tissues and blood when weaning pigs have increased oxidative stress after weaning.

## CONCLUSION

Dietary 25-OHD_3_ supplementation as a vitamin D source in nursery diets at 1,000 and 2,000 IU/kg diet showed the potential to improve feed intake and growth rate in the late nursery period, increased plasma 25-OHD_3_ concentrations in a dose-dependent manner, and decreased plasma MDA levels in the late nursery period compared with VD_3_ supplementation. The plasma MDA level was correlated with plasma vitamin D status and minimized when the plasma 25-OHD_3_ level reached 23.7 ng/mL, that could be achieved by approximately 1,600 IU/kg 25-OHD_3_ supplementation level. Because the current study only used two 25-OHD_3_ supplementation levels with limited number of pigs, further studies are needed to demonstrate the optimal 25-OHD_3_ supplementation levels with various supplementation levels in a larger scale research setting.

## Figures and Tables

**Table 1:** Diet formulation and calculated chemical composition (as-fed basis)

Ingredients (%)	Phase 1 d 0–14 postweaning	Phase 2 d 14–28 postweaning
Corn	38.90	49.50
Soybean meal (48% crude protein)	26.50	31.50
Whey, dried	5.00	7.50
Oats	2.50	2.50
HP300^1)^	5.00	2.00
Lactose	10.00	0.00
Fish meal	3.00	1.50
Animal plasma	3.00	0.00
Soybean oil	2.40	1.90
Corn starch	1.00	1.00
L-Lysine·HCl	0.13	0.22
DL-Methionine	0.16	0.15
L-Threonine	0.09	0.13
Dicalcium phosphate	0.75	0.55
Limestone	0.97	0.95
Salt	0.25	0.25
Trace mineral mix^2)^	0.15	0.15
Vitamin mix^3)^ (vitamin D free)	0.20	0.20
Total	100.00	100.00
Calculated chemical composition		
Metabolizable energy (kcal/kg)	3,441	3,391
Crude protein (%)	24.10	22.78
SID lysine (%)	1.38	1.28
SID methionine+cysteine (%)	0.84	0.76
Total Ca (%)	0.80	0.70
Total P (%)	0.64	0.58
STTD P (%)	0.40	0.33

1) Hamlet Protein, Findlay, OH.

2) The trace mineral premix supplied the following per kilogram of diet: 33 mg of Mn as manganous oxide, 110 mg of Fe as ferrous sulfate, 110 mg of Zn as zinc sulfate, 16.5 mg of Cu as copper sulfate, 0.3 mg of I as Ca iodate, 0.3 mg of Se as sodium selenite,

3) The vitamin premix supplied the following per kilogram of diet: 11,000 IU of vitamin A, 99 IU of vitamin E, 4.4 mg of vitamin K, 55 μg of vitamin B12, 9.9 mg of riboflavin, 31.9 mg of pantothenic acid, 55 mg of niacin, 0.9 mg of folic acid, 3.9 mg of vitamin B6, 3.1 mg of thiamin, and 0.3 mg of biotin, 600 mg of choline chloride.

SID, standardized ileal digestible; STTD, standardized total tract digestible.

**Table 2:** Growth performance of pigs fed diets supplemented with vitamin D_3_ or 25 hydroxycholecalciferol (25-OHD_3_) in nursery period

	Treatment^1)^					

Vitamin D_3_ (IU/kg)	2,000	0	0		p-value	
25-OHD_3_ (IU/kg)	0	1,000	2,000	SEM	Treatment	D_3_ vs. 25-OHD_3_^2)^
Body weight (kg)						
d 0 postweaning	5.43	5.28	5.26	0.76	0.48	0.25
d 7 postweaning	5.87	5.69	5.87	0.80	0.51	0.56
d 14 postweaning	8.29	8.06	8.22	0.96	0.86	0.69
d 21 postweaning	11.29	11.14	11.26	1.23	0.94	0.83
d 28 postweaning	15.32	15.56	15.70	1.58	0.52	0.31
ADG (kg/d)						
d 0–7 postweaning	0.063	0.058	0.088	0.013	0.30	0.55
d 7–14 postweaning	0.346	0.339	0.336	0.030	0.97	0.81
d 14–21 postweaning	0.428	0.440	0.434	0.043	0.84	0.61
d 21–28 postweaning	0.576	0.632	0.634	0.054	0.12	0.05
d 0–14 postweaning (Phase I)	0.205	0.199	0.212	0.019	0.87	0.97
d 14–28 postweaning (Phase II)	0.502	0.536	0.534	0.047	0.12	0.05
d 0–28 postweaning (overall)	0.353	0.367	0.373	0.030	0.21	0.10
ADFI (kg/d)						
d 0–7 postweaning	0.169	0.155	0.174	0.018	0.30	0.70
d 7–14 postweaning	0.459	0.429	0.423	0.037	0.44	0.22
d 14–21 postweaning	0.701	0.705	0.708	0.064	0.97	0.85
d 21–28 postweaning	0.862^b^	0.932^a^	0.932^a^	0.074	0.03	0.01
d 0–14 postweaning (Phase I)	0.314	0.292	0.298	0.027	0.50	0.28
d 14–28 postweaning (Phase II)	0.782	0.818	0.820	0.068	0.16	0.07
d 0–28 postweaning (overall)	0.548	0.555	0.559	0.046	0.77	0.51
G:F						
d 0–7 postweaning	0.368	0.357	0.519	0.061	0.20	0.39
d 7–14 postweaning	0.754	0.791	0.804	0.046	0.67	0.40
d 14–21 postweaning	0.612	0.623	0.612	0.020	0.85	0.80
d 21–28 postweaning	0.671	0.676	0.680	0.014	0.90	0.69
d 0–14 postweaning (Phase I)	0.652	0.678	0.721	0.042	0.53	0.39
d 14–28 postweaning (Phase II)	0.645	0.653	0.650	0.014	0.89	0.66
d 0–28 postweaning (overall)	0.646	0.660	0.670	0.012	0.45	0.27

n=4 replicate pens per treatment.

1) Treatments: (1) basal diet with 2,000 IU/kg vitamin D_3_ supplementation, (2) basal diet with 1,000 IU/kg 25-OHD_3_ supplementation, and (3) basal diet with 2,000 IU/kg 25-OHD_3_ supplementation.

2) Single degree of freedom contrast (2,000 IU/kg vitamin D_3_ supplementation vs. 1,000 and 2,000 IU/kg 25-OHD_3_ supplementation).

a,b Means with different superscripts within a row differ (p<0.05).

SEM, standard error of the means; ADG, average daily gain; ADFI, average daily feed intake; G:F, gain-to-feed.

**Table 3:** Plasma 25-hydroxycholecalciferol (25-OHD_3_) concentrations (ng/mL) in pigs fed diets supplemented with vitamin D_3_ or 25-OHD_3_ in nursery period

	Treatment^1)^					

Vitamin D_3_ (IU/kg)	2,000	0	0		p-value	
25-OHD_3_ (IU/kg)	0	1,000	2,000	SEM	Treatment	D_3_ vs. 25-OHD_3_^2)^
d 14 postweaning	10.50^c^	15.95^b^	28.83^a^	1.40	0.01	0.01
d 28 postweaning	7.79^c^	14.80^b^	30.08^a^	1.33	0.01	0.01

n=8 replicates per treatment.

1) Treatments: (1) basal diet with 2,000 IU/kg vitamin D_3_ supplementation, (2) basal diet with 1,000 IU/kg 25-OHD_3_ supplementation, and (3) basal diet with 2,000 IU/kg 25-OHD_3_ supplementation.

2) Single degree of freedom contrast (2,000 IU/kg vitamin D_3_ supplementation vs. 1,000 and 2,000 IU/kg 25-OHD_3_ supplementation).

a–c Means with different superscripts within a row differ (p<0.05).

SEM, standard error of the means.

**Table 4:** Plasma superoxide dismutase activity, total antioxidant capacity, and malondialdehyde level in pigs fed diets supplemented with vitamin D_3_ or 25 hydroxycholecalciferol (25-OHD_3_) in nursery period

	Treatment^1)^					

Vitamin D_3_ (IU/kg)	2,000	0	0		p-value	
25-OHD_3_ (IU/kg)	0	1,000	2,000	SEM	Treatment	D_3_ vs. 25-OHD_3_^2)^
Superoxide dismutase (U/mL)						
d 14 postweaning	3.89	4.42	4.43	0.38	0.53	0.27
d 28 postweaning	4.22	3.95	4.23	0.45	0.88	0.81
Total antioxidant capacity (mM trolox equivalents)						
d 14 postweaning	2.82	3.59	3.09	0.34	0.29	0.23
d 28 postweaning	4.82	6.04	5.28	0.70	0.48	0.34
Malondialdehyde (μM)						
d 14 postweaning	8.41	9.36	9.72	0.62	0.34	0.16
d 28 postweaning	14.24^b^	10.40^ab^	7.94^a^	1.53	0.03	0.02

n=8 replicates per treatment.

1) Treatments: (1) basal diet with 2,000 IU/kg vitamin D_3_ supplementation, (2) basal diet with 1,000 IU/kg 25-OHD_3_ supplementation, and (3) basal diet with 2,000 IU/kg 25-OHD_3_ supplementation.

2) Single degree of freedom contrast (2,000 IU/kg vitamin D_3_ supplementation vs. 1,000 and 2,000 IU/kg 25-OHD_3_ supplementation).

a,b Means with different superscripts within a row differ (p<0.05).

SEM, standard error of the means.
